# Infection of Slugs with Theronts of the Ciliate Protozoan, *Tetrahymena rostrata*

**DOI:** 10.3390/microorganisms9091970

**Published:** 2021-09-16

**Authors:** Ruth E. Haites, Anne E. Watt, Derek A. Russell, Helen Billman-Jacobe

**Affiliations:** Asia-Pacific Centre for Animal Health, Melbourne Veterinary School, Faculty of Veterinary and Agricultural Sciences, University of Melbourne, Melbourne 3010, Australia; ruth.haites@unimelb.edu.au (R.E.H.); watta@unimelb.edu.au (A.E.W.); derek.russell@unimelb.edu.au (D.A.R.)

**Keywords:** parasitic ciliate, theronts, trophonts, cysts, mollusks

## Abstract

*Tetrahymena rostrata* is a free-living ciliated protozoan and is a facultative parasite of some species of terrestrial mollusks. It is a potential biopesticide of pest slugs, such as the grey field slug, which cause considerable damage to crops. *T. rostrata* has several developmental forms. Homogeneous preparations of the feeding stage cells (trophonts) and excysted stage cells (theronts) were compared for their ability to infect and kill *Deroceras reticulatum* slugs. Theronts were more effective and remained viable and infective, even after prolonged starvation.

## 1. Introduction

*Tetrahymena rostrata* (Kahl, 1926; Corliss 1952) (Ciliophora: Oligohymenophorea) has been shown to infect and kill species of land slugs and snails and has been proposed as a possible biological control agent of pest mollusks. This paper examines the infectivity of different life stages of *T. rostrata* to terrestrial mollusks with a view to advance these prospects.

**Figure 1:**
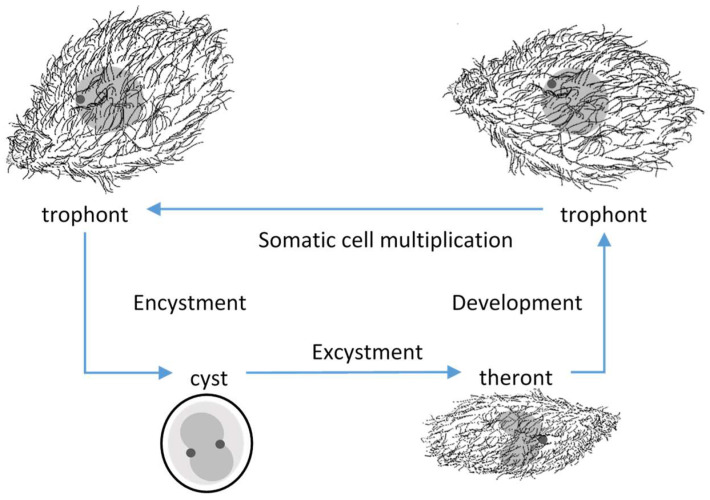
Simplified schematic of the *Tetrahymena rostrata* lifecycle showing trophonts, cysts and theronts, which are distinguished by their cellular morphology and the arrangement of the macronucleus (grey) and small micronucleus (dark grey).

*Tetrahymena rostrata* was first isolated from environmental samples, putrescent *Glyceria* [1], moss [2] and leaf litter [3]. Four developmental stages of *T. rostrata* could be distinguished; trophonts, tomonts, tomites and theronts [2,3] (Figure 1). The pyriform, free-swimming, feeding, trophonts occur when food sources are available. Trophonts are highly plastic and multiply by mitosis. In the absence of food, trophonts become fast-swimming tomites and secrete mucins, which form a soft capsule around tomonts, which develop into cysts in which meiosis occurs. Excystment occurs spontaneously or in response to the availability of nutrients. Theronts, which emerge from the cyst capsules, are attracted to proteinaceous materials and convert into trophonts after feeding [3,4]. *T. rostrata* can be free living and can be grown in an axenic culture. However, natural infections have been recorded in enchytraeid worms, *Enchytraeidae* [3], *Deroceras reticulatum* (Müller 1744) slugs [5,6,7] and some species of snail [4,7,8]. Experimental infections of *D. reticulatum* with *T. rostrata* trophonts suggested that the ciliates enter the slug via a pouch under the mantle and then migrate primarily to the renal tissues and later to other organs, resulting in the death of the host [5].

The life cycles of many other ciliates which undergo encystment, have been described but the role of the theronts has received relatively little attention. This is possibly due to the technical difficulty of separating theronts and trophonts in cultures. The best studied theronts are from the commercially important fish parasites, *Ichthyophthirius multifiliis* (Fouquet, 1876) (Ciliophora; Oligohymenophorea) [9,10] and *Cryptocaryon irritans* (Brown, 1951) (Ciliophora: Prostomatea) [11,12] and the bivalve parasite *Ophryoglena hemophaga* (Molloy, Lynn and Giamberini, 2005) (Ciliophora: Ophryoglenidae) [13], whose theronts are short-lived if they do not find a suitable host. In contrast, the free-living aquatic ciliates, *Holophrya teres* (Ehrenberg, 1834) (Ciliophora: Prostomatea) and *Prorodon* *aklitolophon* ((Hiller and Bardele, 1988) (Ciliophora: Prostomatea) form reproductive cysts, which release theronts that can last for several days [14,15]. However, if food does not become available, the theronts form a thin mucous coating and re-encyst. Successive rounds of spontaneous excystment and re-encystment result in gradual reduction in the size of the theronts until they are too small to feed. In the case of *P. aklitolophon*, theronts survived under continual starvation for up to 8 weeks [15].

The only published experimental infections with *T. rostrata* were performed using *D. reticulatum,* and those studies utilized trophonts derived from axenic cultures [5,16]. In this paper, we show that *T. rostrata* theronts are more effective at establishing lethal infections of slugs than are trophonts, and that theronts can be long-lived in the absence of nutrients.

## 2. Materials and Methods

### 2.1. Tetrahymena rostrata

*T. rostrata* isolate TRAUS was originally isolated from the egg of a *D. reticulatum* slug collected in Melbourne, Australia, in 2014 [17]. Cultures of *T. rostrata* were maintained at 20 °C in the dark in sterile PPYE medium (0.5% *w/v* proteose peptone (Oxoid, LP0085), 0.5% *w/v* yeast extract (Oxoid, LP0021) and 0.125% *w/v* glucose), and subcultures were performed fortnightly. Cultures prepared for encystment were grown in sterile PP medium (1% *w/v* proteose peptone (Oxoid, LP0085), and 0.125% *w/v* glucose prior to nutrient starvation. The taxonomic systematics of Lynn, 2008 were followed when referring to ciliate species [18].

### 2.2. Preparation of Theronts

The encystment buffer was prepared by mixing 50 g of medium grade composted pine bark (Australian Growing Solutions) in 1.2 L of MilliQ ultrapure water for 15 min. The mixture was sieved, and the flow-through was centrifuged at 300× *g* for 10 min to remove large solids. The decanted supernatant was sterilized by autoclaving at 121 °C for 20 min. N-(2-Hydroxyethyl)piperazine-N′-(2-ethanesulfonic acid) (HEPES) pH7 (NaOH) was added to a final concentration of 10mM prior to use.

*T. rostrata* cells from exponentially growing PP cultures were collected by centrifugation (800× *g*, 10 min) and washed with 10 mM HEPES pH 7 NaOH, and then resuspended in encystment buffer at a final density of 1–3 × 10^4^ cells/mL. Cells were placed into 6-well plates (Greiner Bio-one Cat No. 657 185) in 3 mL aliquots or in T150 vented tissue culture flasks in 100 mL aliquots and incubated at 26 °C to allow cysts to form. After 1–3 days at 26 °C, the cyst cultures were transferred to 20 °C to excyst and release the theronts. Excystment started on day 5 and peaked at day 7, with 85 to 90% of the cysts being excysted. Theronts were stored in the encystment buffer at 20 °C until use.

Preparations of theronts, free from unexcysted cells, were used to infect slugs for histology. This involved excystment as above, followed by centrifugation at 300× *g* for 10 min to collect the cells and debris into a pellet, after which the tubes were left undisturbed for 2 h to allow the theronts to swim upwards and be harvested from the supernatant with a pipette. Trophonts used for the challenge experiments were derived from cysts and cultured in PPYE for 4 days to stimulate excystment and conversion to trophonts. Trophonts were washed free of media using 10 mM HEPES pH7 NaOH immediately before use in a slug challenge experiment. The numbers of trophonts and theronts in the cultures were determined, using a Fuchs–Rosenthal counting chamber with a Leica DMLS light microscopy, and adjusted to the desired concentration by harvesting the cells by centrifugation (800× *g*, 10 min) or dilution in 10 mM HEPES pH7 NaOH.

Wet mounts and fixed Giemsa stained cells were imaged with a Leica DMLS light microscopy and used to confirm cyst, theront and trophont forms. Viable counts were determined using most probable number method, using PPYE as the medium [19].

### 2.3. Dose and Lethality Experiments

Laboratory-reared *D. reticulatum* were maintained as previously described [20]. Young slugs, ~0.8–1 cm long, were selected for all experiments. Each slug was housed in a 25 mL tube containing 3 g of moist potting soil (Plugger 111-Seedraising Mix). The tubes were placed in a humidified box and kept in a controlled environment at 17 °C with a 12 h photoperiod. There were 20 slugs per treatment. The slug’s grazing activity and mortality was assessed daily or weekly, depending on the experiment. Each slug was provided with Chinese cabbage for food, and the cabbage was replaced as required. For dose experiments, 20 replicates of individually housed *D. reticulatum* slugs were exposed to 100,000, 30,000, 10,000, 3000, 1000 or zero trophonts or theronts. The testing of starved theronts was performed, using a dose of 10^5^ theronts, which had all excysted over the preceding 7 days and were used immediately or kept in the encystment buffer at 20 °C until use. Kaplan–Meier plots and analyses were performed in GraphPad Prism 9.0.1.

To examine behavioral changes and histology in experimental infections of *D. reticulatum,* slugs ~1 cm long, were placed individually in 5 cm diameter lidded plastic pots lined with damp filter paper. Half a circular piece of cabbage 2.3 cm in diameter was added to each container. Treated slugs received 8.4 × 10^3^ theronts applied to the filter paper and food. Control slugs were exposed to a mock inoculum of the same volume of buffered solution of encystment buffer. The slugs were monitored daily for early signs of infection, such as a swollen mantle, inability to extend the superior tentacles and reduced locomotion. Reduced extension of the superior tentacles commonly occurred in the days preceding death and was an indication that a slug had a lethal infection. These criteria were used to select moribund slugs for histology to determine the site of infection. *D. reticulatum* slugs were exposed to *T. rostrata* theronts as above and, after 7 days of exposure, 10 slugs from the control group and 26 moribund slugs from the theront-exposed group were selected and euthanized by submersion in soda water, fixed in neutral buffered formalin for 48 h and then transferred to 70% ethanol. The fixed samples were embedded in paraffin wax and sectioned transversely in sections 5 µm thick, which were then were mounted on glass slides and stained with haematoxylin and eosin (H & E). The slides were examined, using a Leica DMLS light microscope. Statistical analysis was performed using GraphPad Prism version 9.0.1.

## 3. Results

### 3.1. More Slugs Exposed to Theronts Die Than Those Exposed to Trophonts

**Figure 2:**
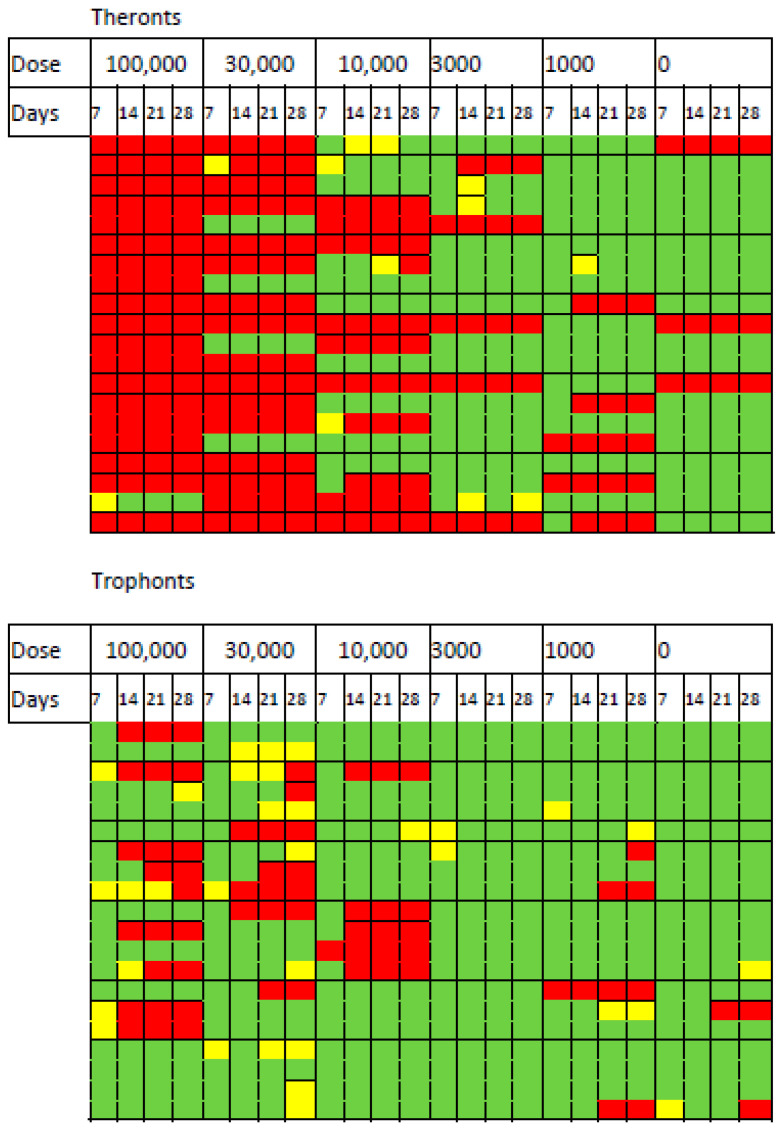
Heatmap showing response of slugs to exposure to different doses of *Tetrahymena rostrata* theronts and trophonts over 28 days of observation. Inhibition of feeding (yellow), death (red) or survival (green). Lines within treatments are for individual slugs.

*D. reticulatum* slugs were exposed to different doses of *T. rostrata* theronts and trophonts, and their survival was monitored over 28 days. The mortality and effects of exposure on grazing are shown in Figure 2. The impact of the theronts was apparent after 7 days of exposure. All of the slugs exposed to the highest dose of theronts (100,000 cells) were affected and 19/20 died within 7 days. For the 30,000 theront treatment, 16/20 slugs died. The LD_50_ was calculated by Logit and Probit analysis based on the responses within the first 7 days. The LD_50_ for theronts was 15,630 (Logit) and 17,321 (Probit).

The same doses of trophonts had less effect on the slugs. At the highest dose of trophonts (100,000), 10/20 slugs were affected by the end of the experiment (9 dead, 1 not eating). Exposure to 30,000 trophonts results in a similar number of deaths, but there were more affected (7 not feeding, 7 dead). Several trophont-exposed slugs stopped feeding for some time before death, suggesting that the course of infection was slower than in theront treated slugs. Insufficient numbers of trophont-exposed slugs died to allow calculation of the LD_50_.

**Figure 3:**
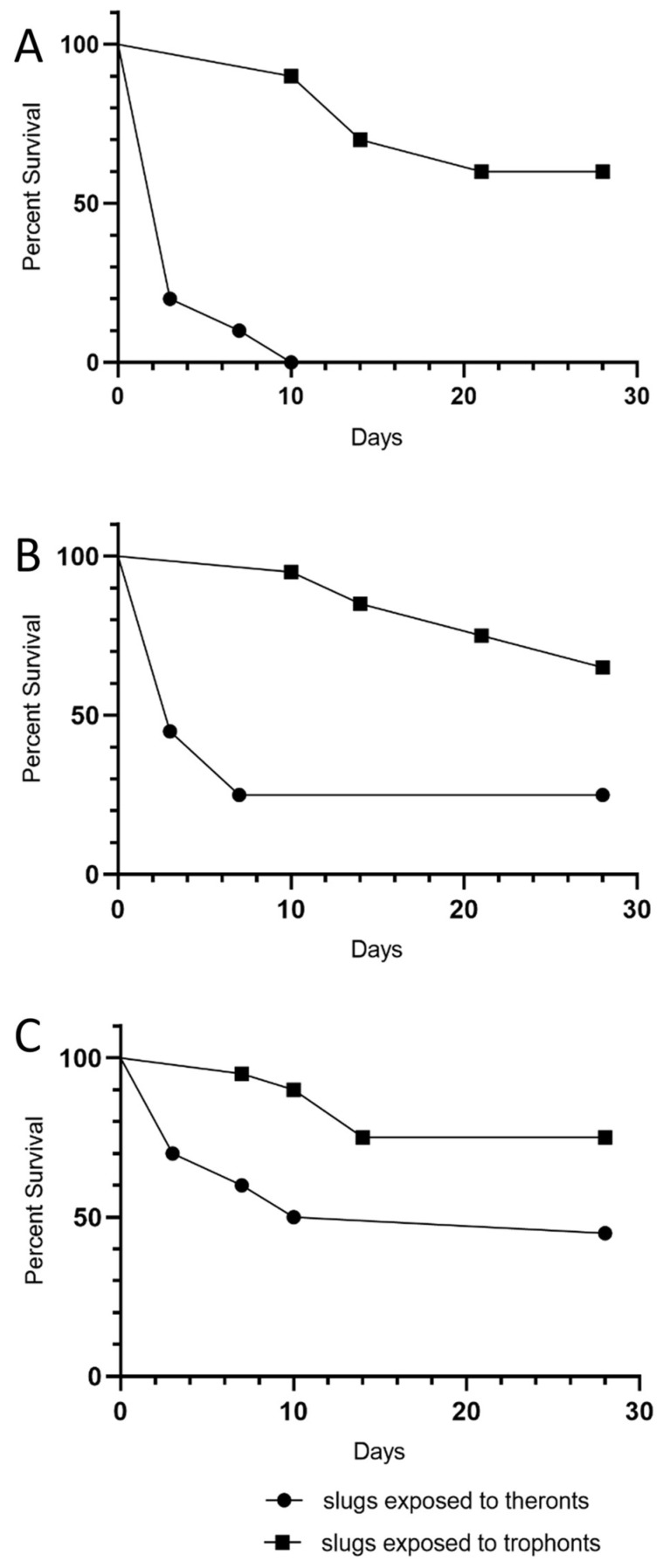
Kaplan-Meier survival plots of *Deroceras reticulatum* slugs exposed to different doses of *T. rostrata* theronts and trophonts. Doses were (**A**) 100,000, (**B**) 30,000 and (**C**) 10,000 theronts or trophonts, respectively (20 slugs per treatment).

The data suggest that the effect of the theronts occurred soon after the slugs were exposed. In contrast, slugs exposed to trophonts were more likely to have stopped eating for several days before dying (Figure 2). Mortality over 28 days was considerably greater in slugs exposed to theronts than trophonts (Figure 3 and Table 1).

### 3.2. Starved Theronts Are Long Lived

**Figure 4:**
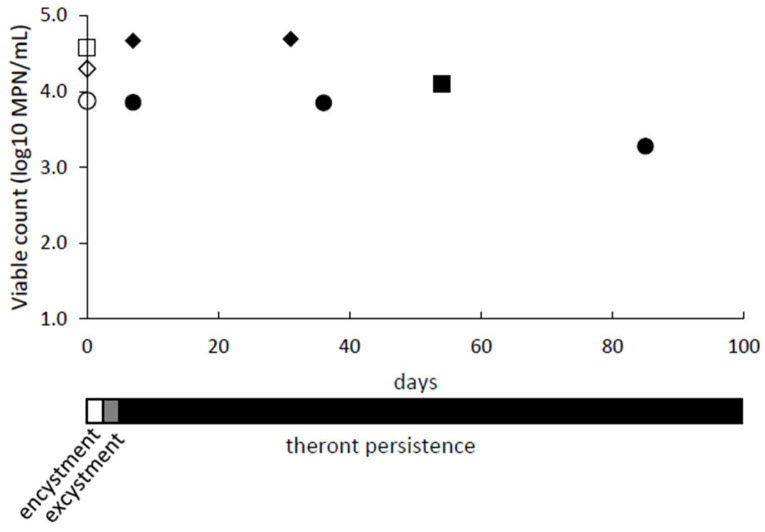
Theronts persist under starvation conditions. Trophonts cultures were encysted in encystment buffer at 26 °C, resulting in cyst suspensions on days 1 and 2. The cysts were transferred to 20 °C, and excystment of theronts was completed by day 7. They were then maintained under starvation conditions for a further 24, 47, or 78 days. The viable counts (log10 MPN/mL) are shown for the trophonts on day 0 (empty symbols) and theronts (filled symbols) for three experiments (circles, diamonds and squares). A summary timeline for encystment, excystment and theront persistence is given.

We observed that theronts remained viable when stored for prolonged periods under starvation conditions in encystment buffer. Populations of theronts in encystment buffer were monitored for up to 85 days by determining viable counts, using Most Probable Number (MPN) assays (Figure 4). The MPN assay determines the number of viable cells present, which could multiply and establish a trophont culture. The culture was monitored by observing and counting cell morphology, as the cells changed from trophonts (day 0) through to cysts during starvation (day 1 and 2) and then theronts after excystment (day 5 to 7). The excysted cells were confirmed to be theronts by Giemsa staining and showed the characteristic bi-lobed macronucleus and a micronucleus. The starved theronts did not become noticeably smaller over time, and there was no evidence of re-encystment. Under equivalent conditions (nutrient starvation, 20 °C), the trophonts developed into cysts and, after about a week, the cysts released theronts.

### 3.3. Starved Theronts Can Kill Slugs

**Figure 5:**
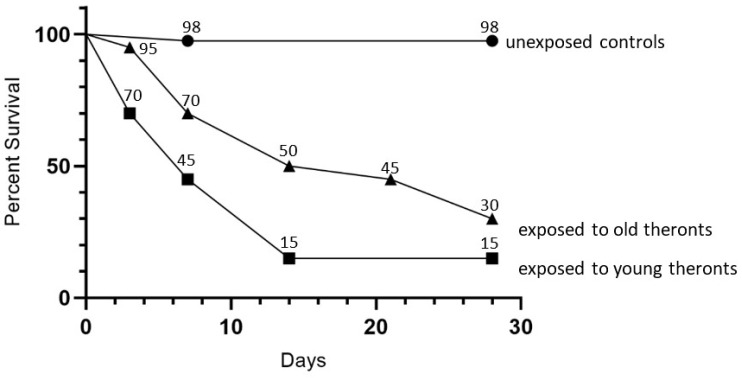
Kaplan–Meier survival plots of *D. reticulatum* slugs exposed to “young” newly excysted theronts, “old” 43-day stored theronts or untreated controls. n = 20 per treatment, n = 40 controls.

The infectivity of starved theronts was determined in a slug challenge, using *D. reticulatum*, with 20 slugs treated with 10^5^ theronts, which were excysted in encystment buffer at 20 °C, then used immediately or stored for a further 43 days, plus 40 untreated controls. The older theronts killed 70% of slugs by 28 days, as compared to the efficacy of newly excysted theronts, which killed 85% of the slugs (Figure 5). The log-rank (Mantel–Cox) test showed that curves for the young and old theront treatments were not different (*p* = 0.57), whereas the curves for slug survival for both the young and old theront treatments were significantly different from the control groups (*p* < 0.0001).

### 3.4. Infected Slugs Become Moribund

**Figure 6:**
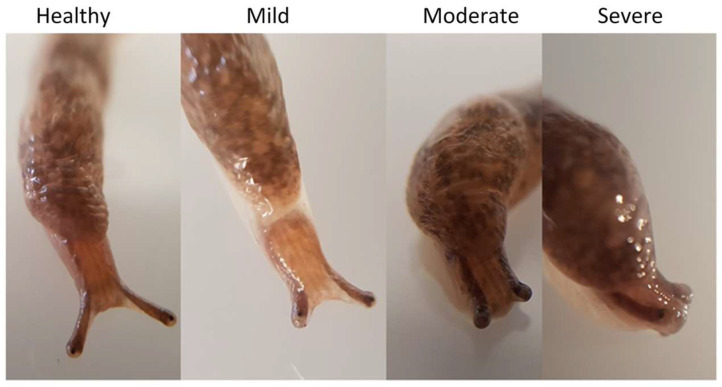
Slugs exposed to theronts displaying superior tentacle impairment: healthy slug not exposed to theronts; mild, moderate and severely impaired slugs after exposure to theronts.

**Figure 7:**
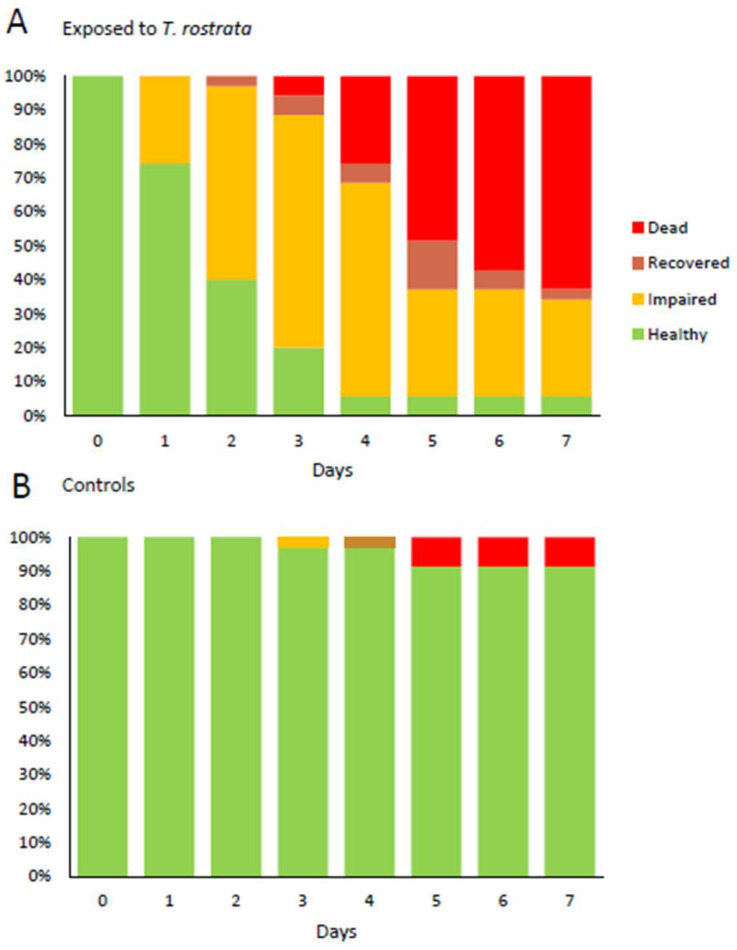
Health status of *D. reticulatum* over 7 days (**A**) after exposure to *T. rostrata* theronts and (**B**) without exposure to *T. rostrata.* Slugs were assessed as “impaired” if they displayed reduced ability to extend the superior tentacles and as “recovered” if they displayed normal tentacle movement on days after being assessed as impaired.

In order to study the progress of infection, *D. reticulatum* slugs were exposed to pure preparations of theronts. These differed from the preparations in the previous experiments only by the factor that there were no unexcysted or dead cells present. The behavior of the slugs was monitored daily. In each of three experiments, slugs exposed to theronts rapidly showed signs of reduced mobility of their superior tentacles, and this sign was most evident in the day or two before death (Figure 6 and Figure 7).

### 3.5. T. rostrata Invades Multiple Organs of D. reticulatum

**Figure 8:**
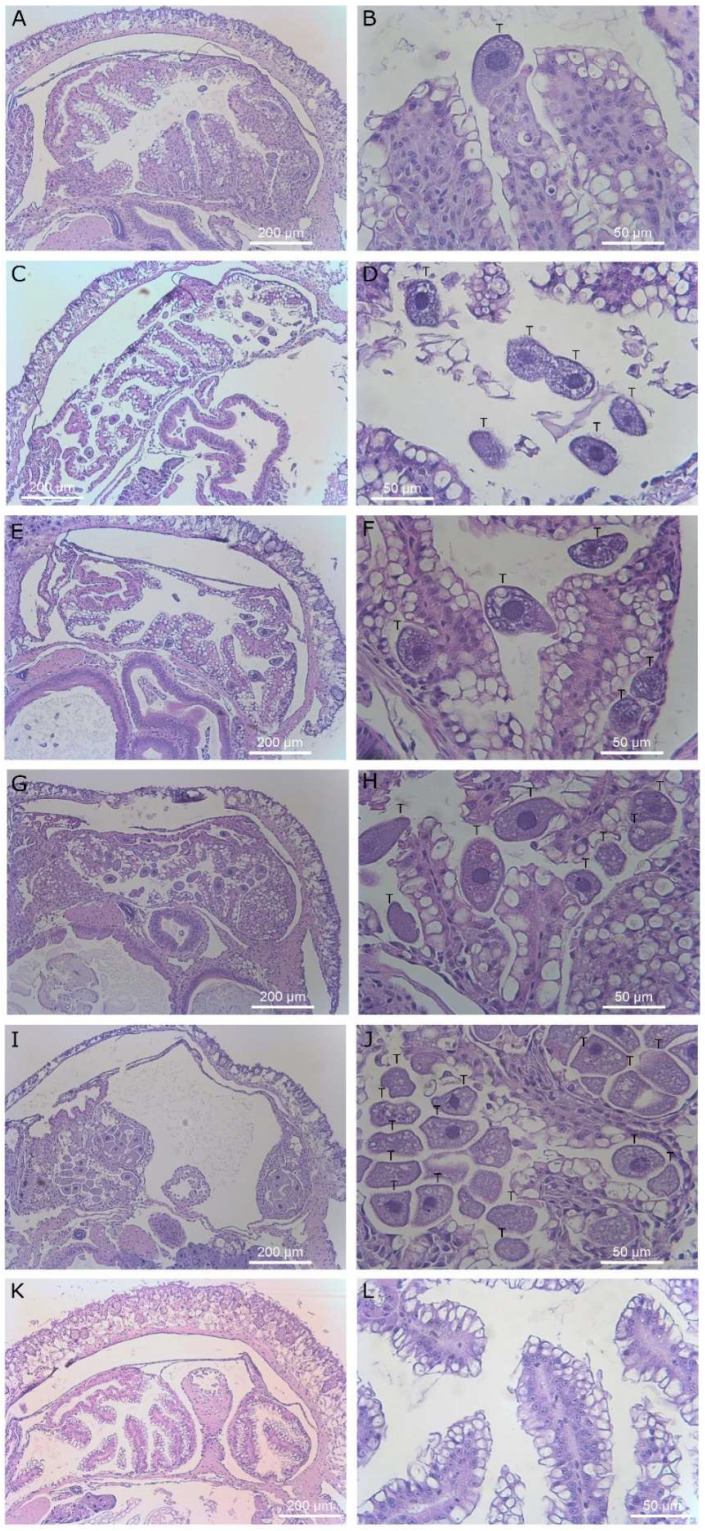
Histological sections of slug kidney. Magnification ×40 (left column) and ×400 (right column). Images (**A**–**H**) display slugs exposed to *T. rostrata*. (**A**–**H**) shows ciliates free swimming within the saccular portion of the kidney along with several ciliates encapsulated in granulomas and others grazing on vacuolar cells or encapsulated, (**I**–**L**) show the saccular portion of the kidney of a healthy control slug, showing healthy kidney structure. Ciliates are marked with a “T” above the cells in panels (**B**,**D**,**F**,**H**,**J**).

**Figure 9:**
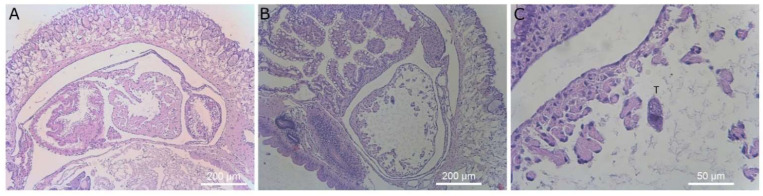
Histological sections of the pulmonary region. Image (**A**) shows the pulmonary region of a healthy control slug. Images (**B**) (×40 mag) and (**C**) (×400 mag) show a single ciliate in the heart and an enlarged chamber of the heart of a slug exposed to *T. rostrata*. Ciliates are marked with a “T” above the cell in panel (**C**).

**Figure 10:**
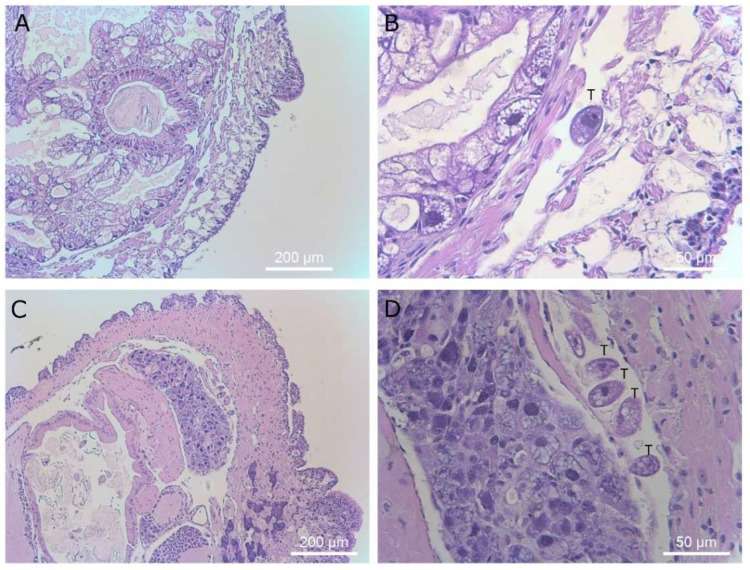
Histological sections of slugs showing ciliates in the muscle tissue between the skin and body cavity of the slug. Magnifications are ×40 (panel **A** and **C**) and ×400 (panel **B** and **D**). Ciliates are marked with a “T” above the cells in panels (**B**,**D**).

**Figure 11:**
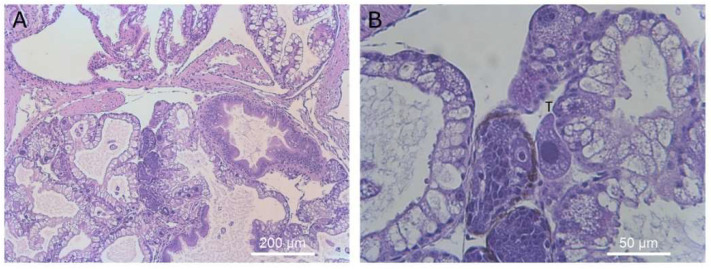
Histological section of reproductive organs showing a single ciliate adjacent to the developing gonad. Magnification ×40 (**A**) and ×400 (**B**). Ciliates are marked with a “T” above the cell in panel (**B**).

**Figure 12:**
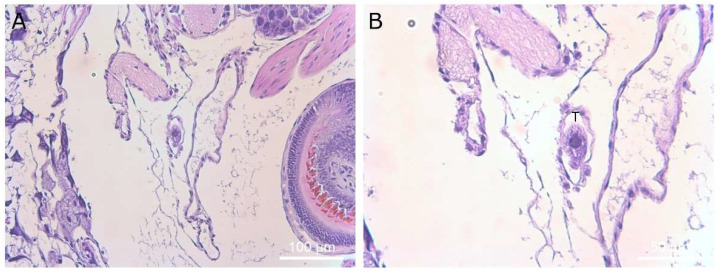
Histological section showing a single ciliate in an artery of the slug. Magnification ×40 (**A**) and ×400 (**B**). Ciliates are marked with a “T” above the cell in panel (**B**).

**Figure 13:**
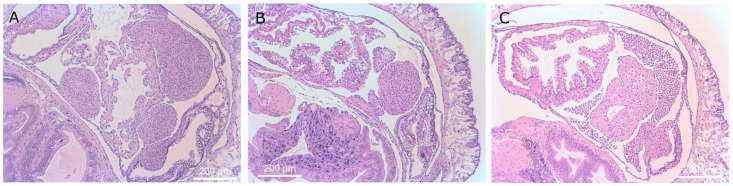
Histological sections of slugs showing masses of inflammatory cells. Images (**A**,**B**), ×40 mag. These structures are formed from the epicardial cells of the heart and kidney. Image (**C**) shows aggregating hypertrophic amoebocytes, ×40 mag.

*D. reticulatum* slugs that were exposed to theronts and exhibited superior tentacle impairment were selected for histological examination. Ciliates were found in the tissues of 20 out of 26 slugs. This may be an underestimation because the presence of one or more ciliates was only recorded if the cell shape, presence of cilia and a macronucleus were observed, and some sections may have missed the macronucleus. There were no apparent pathological changes in the tentacles or occurrence of ciliates in the vicinity of the tentacles. Ciliates were found predominantly in the kidney (16/20 slugs) (Figure 8), lungs (11/20 slugs) (Figure 9), muscle (9/20 slugs) and connective tissue (5/20 slugs) (Figure 10). Occasional ciliates were observed in the heart, arteries, pneumostome passage and hepatopancreas (Figure 11 and Figure 12). Some areas of inflammation were apparent (Figure 13). The nuclear arrangement of the parasitic *T. rostrata* was consistent with the trophont form, i.e., one micronucleus and one ovoid macronucleus.

## 4. Discussion

There are eight classes of ciliates and six species of *Tetrahymena* that can form resting cysts [18]. The triggers for ciliate excystment are highly variable [21]. For *T. rostrata*, triggers of excystment include media containing peptone and tryptone or milk, kidney extract, mollusk mucous and changes in temperature or salinity [3,4,22]. Spontaneous excystment of reproductive cysts is frequent, indicating that metabolic triggers also exist [4,21,22]. Our method of preparation of cysts results in highly efficient, synchronized encystment with almost 100% of cells encysting. Synchronized excystment results in almost pure populations of theronts, which can be further separated from unencysted or dead cells, if required. These methods enabled us to compare the performance of trophonts and theronts in slug infection experiments.

Previous studies have shown that *T. rostrata* trophonts are capable of infecting and killing *D. reticulatum* slugs [5], and naturally-infected specimens of a variety of slugs and snails have been described. Clearly, *T. rostrata* trophonts are infective. In edaphic environments, ciliates, such as *T. rostrata,* would be expected to occur as trophonts in a growth-division cycle when moisture and nutrients are not limiting and in an encystment cycle when conditions are less favorable.

Our experiments showed that exposure to theronts was more likely to result in rapid slug death than exposure to the same dose of trophonts. Tests with trophonts may have resulted in the same eventual death rate if the experiment had gone long enough. Following infection and initial feeding, the theronts convert to trophonts, which cause tissue damage and ultimately kill the slugs. This may be an important consideration if *T. rostrata* is to be used as a biopesticide for the control of pest slugs. We used young slugs in these studies and housed them individually. Slugs are known to exhibit huddling behavior when they are threatened with dehydration [23]. It would be interesting to measure the efficacy of theronts against different sizes of slugs, housed singly and in groups.

Soil ciliates are expected to occur largely as cysts in soil and to enter the vegetative growth phase periodically when conditions are favorable. The extensive studies of the late W. Foissner are testament to the persistence of cysts in soil (for example, [24]). The longevity and infectivity of starved *T. rostrata* theronts described here was unexpected. We are cautious about extrapolating from controlled laboratory conditions to environmental situations. However, it may be possible that, under some conditions, theronts might persist for prolonged periods until they encounter a suitable host mollusk or other nutrient source. In the slug exposure experiment, the trophonts and theronts may have several responses. They may remain in their developmental form and infect a slug or remain in the substrate. Alternatively, they may die or change their developmental form. We know at least some theronts developed into trophonts, as these are what we see in the histological sections. Trophonts could encyst and release theronts. It is most likely that all of these changes occur within the population depending on the microniches that cells encounter.

The occurrence of reduced mobility of the superior tentacles was a strong indicator of the subsequent death of slugs exposed to theronts. There was no histological evidence of damage to the superior tentacles, nor were any ciliates identified in the area. The ciliates were found predominantly in the kidney and pulmonary chamber. The ciliates found in the kidney appeared to be multiplying, as dividing cells were observed. It is possible that *T. rostrata* is attracted to metabolites concentrated in the kidney, such as urea or purines.

The presence of individual ciliates in places other than the kidney in these slugs suggests that they were mobile within the slugs. The histological sectioning also provides insight into the life stage of the ciliates inside the slugs. Theronts have a characteristic lobulated macronucleus, whereas the ciliates that were present in the sectioned slugs showed the characteristic form of trophonts with a single round macronucleus and associated micronucleus. This was not unexpected, as the transformation from theront to trophont in the presence of suitable food takes only a few hours, and the slugs were sectioned 7 days after they were exposed to theronts. We placed 6-week-old theronts onto autoclaved slug tissues and observed them immediately feeding, with the majority of theronts converting to trophonts within 4 h (data not shown).

Trophonts were most abundant in the saccular portion of the kidneys and were associated with damage to the renal cells, leaving the basal cells intact. These finding reinforce those of Brooks [5] who concluded that the mechanism for *T. rostrata* in the destruction of the kidney was enzymatic, mechanical, or both [5]. It is likely that slugs with renal tissue damage would have impaired ability maintain their water balance and the behavior observed, i.e., contracted postures with retracted tentacles, is consistent with slugs that are dehydrated [23].

Ciliates were seen in the muscle, between the skin and muscle layers of the slug and in the interstitial space. The ciliates in the muscle appeared smaller than those in the kidney, possibly because of space constraints or because those niches are not so conducive to feeding and growth. Ciliates were also found individually adjacent to the developing gonads of some slugs. However, these slugs used were young, and the gonads were not fully developed. Infection of the albumen gland was reported in naturally infected D*. reticulatum* and was associated with transovarial transmission [5]. It is worth noting that the *T. rostrata* isolate used in these studies was originally isolated from the albumen of a *D. reticulatum* egg laid by an infected parent. It is surprising that *T. rostrata* encyst inside *D. reticulatum* eggs [5]. This may be an adaptation, with the more infective theronts being released when eggs are ruptured. This would be an ideal adaptation for this species because the infective theronts would have ready access to neonates in the egg cluster. However, in our experience, it is rare to find infected eggs, even in infected laboratory- reared *D. reticulatum*. Neither trophonts nor theronts are able to penetrate eggs that have already been laid.

Some ciliates appeared to be trapped within groups in abnormally proliferating renal cells, possibly hypertrophic fibroblasts and amoebocytes. These granulomata formed around individual and groups of ciliates. Three of the slugs exposed to theronts and examined though histology, displayed abnormal changes in the heart. The pericardial cavities of these three slugs were filled with cells and hypertrophic amoebocytes. These cellular masses appeared identical to those seen by Brooks [5], who described them as being composed of hypertrophic amoebocytes surrounded by a layer of epicardial cells.

No attempt was made to determine the route of entry of the theronts into the slugs. It was suggested that they enter via the integumental pouch under the mantle. However, we did not observe any trophonts in the mantle cavity. We did detect ciliates in the pneumostome passage passing under the pulmonary chamber. The tissue walls are very thin in this area and would seem to be vulnerable to mechanical damage from the ciliates, allowing access to the pulmonary chamber. We did not observe any direct evidence of trophonts squeezing between epithelial cells, as occurs with *I. multifiliis* theronts [10]. The ingestion of ciliates remains a possibility as the route of infection, as ciliates were also found in the hepatopancreas (also known as the digestive gland). However, this is less likely, as there were very few *T. rostrata* ciliates seen in this gland, possibly due to size limitations for particles passing into the gland. Once at the hepatopancreas, the ciliates would need to cause mechanical damage to rupture the gland wall. Due to the small number of ciliates seen in this organ and the surrounding tissues and arteries, this remains unlikely.

The method for preparation of 100% theront cultures and the behavioral signs of infection are useful tools for further studies on the infection process.

## Figures and Tables

**Table 1:** Log rank analysis of difference between Kaplan–Meier survival plots of slugs exposed to doses of theronts, trophonts or untreated controls.

Dose	Theronts vs. Control	Trophonts vs. Control	Theronts vs. Trophonts
100,000	<0.0001 (****)	0.0115 (*)	<0.0001 (****)
30,000	<0.0001 (****)	0.0288 (*)	0.0008 (**)
10,000	<0.0001 (****)	ns	0.0279 (*)
3000	0.0016 (**)	ns	0.0182 (*)
1000	0.0017 (**)	0.0055 (**)	ns

*p* value summary, ns—not significant, * *p* > 0.05, ** *p* ≤ 0.01, *** *p* ≤ 0.001, **** *p* ≤ 0.0001.

## Data Availability

Data is contained within the article.
